# Patients’ experiences of responding to a Patient-Reported Outcome Measure for palliative care: a mixed method study

**DOI:** 10.1007/s11136-025-04006-w

**Published:** 2025-06-14

**Authors:** Jahan Shabnam, Mette Raunkiær, Maiken Bang Hansen, Mogens Grønvold, Anders Løkke, Edina Nikolett Barna, Camilla Lykke, Tina Broby Mikkelsen, Cecilie Lindstöm Egholm

**Affiliations:** 1 REHPA, The Danish Knowledge Centre for Rehabilitation and Palliative Care, Odense University Hospital, Nyborg, Denmark; 2 Department of Clinical Research, University of Southern Denmark, Odense, Denmark; 3 Palliative Care Research Unit, Department of Geriatrics and Palliative Medicine GP, Bispebjerg and Frederiksberg Hospital, University of Copenhagen, Copenhagen, Denmark; 4 Department of Medicine, Vejle, Lille Baelt Hospital, Vejle, Denmark; 5 Department of Regional Health Research, University of Southern Denmark, Odense, Denmark; 6 Department of Oncology and Palliative Care, North Zealand Hospital, The Section of Palliative Medicine, Department of Oncology, Rigshospitalet, Copenhagen University Hospital, Hillerød, Denmark

**Keywords:** Patient reported outcome measures (PROM), Palliative care, Patients’ experiences, Mixed method

## Abstract

**Background:**

Worldwide, there is growing interest in using Patient-Reported Outcome Measures (PROM) in palliative care. The Danish Health Data Authority has developed a new PROM called PRO-Pall, which was launched for nationwide use in patients with heart, lung, and kidney diseases, as well as cancer.

**Aim:**

To explore patients’ experiences of responding to the PRO-Pall and using it in a consultation about palliative care needs in non-specialised palliative care settings.

**Methods:**

This is a multicentre, mixed-methods study combining a quantitative approach using structured questionnaires (*n* = 270) and a qualitative analysis based on semi-structured interviews (*n* = 17). The quantitative survey included items assessing relevance, ease of use, and perceived benefits of PRO-Pall in preparing for consultations. Qualitative data collection involved interviews focusing on patients’ experiences with PRO-Pall and its integration into clinical discussions. Quantitative data were analysed descriptively as numbers (n) and proportions (%), while qualitative data were analysed using thematically using to identify key themes.

**Results:**

A total of 270 patients completed the survey, and 17 patients participated in interviews. The analysis revealed that the patients evaluated the PRO-Pall positively. The patients strongly agreed or agreed that the PRO-Pall was relevant (85%), easy to fill out (85%), helped to become aware of symptoms (61%) and a good way to prepare for the consultation (70%). The qualitative findings further supported these results, highlighting that patients found PRO-Pall relevant, appropriate, and convenient. Participants emphasized that PRO-Pall responses should be actively discussed during consultations with healthcare professionals to enhance its impact. Additionally, they noted that the timing of completing PRO-Pall was crucial, as patients’ conditions fluctuate over time, potentially influencing their responses.

**Conclusion:**

Most patients found the PRO-Pall relevant, appropriate, and easy to fill out. However, its effectiveness depends on healthcare professionals actively integrating patient responses into consultations. Otherwise, it would serve merely as documentation rather than an avenue for patients to discuss their concerns.

**Supplementary Information:**

The online version contains supplementary material available at 10.1007/s11136-025-04006-w.

## Background and aim

Patient-reported outcome measures (PROMs) assess patients’ subjective perceptions of health status and outcomes and provide complementary information to objective measurements [1, 2]. There is a growing interest in understanding the use of PROMs in palliative care [3–5], and the European Association for Palliative Care issued a White Paper in 2016 promoting PROMs in palliative care [6]. Existing evidence indicates that PROMs can help identify unmet needs, provide feedback on treatment effects, and improve communication between patients and healthcare professionals [4, 5, 7, 8]. Although PROMs hold potential, appropriate implementation is crucial to achieve the expected benefits of PROMs. Previous research has shown that several factors can positively or negatively affect the use of PROMs. Promoting factors include establishing skilled coordinators, promoting local ownership, and competence development for healthcare professionals who utilise the tool [4, 7]. Further, research on PROMs often emphasises healthcare professionals’ perspectives since they can support or hinder application [9–11]. Exploring patients’ experiences with PROMs in non-specialized palliative care settings has been rare. However, it is important to consider patient perceptions as they may influence implementation [12, 13].

The Danish Health Data Authority carried out an initiative to develop and use PROM within palliative care by creating a national, standardised PROM for non-specialised palliative care settings (hereafter, ‘PRO-Pall’) [14–16]. The PRO-Pall was launched in 2023 to be implemented in palliative care in primary care and hospitals for patients with heart-, lung- and kidney disease or cancer [15].

The PRO-Pall was developed for non-specialised palliative care and takes a holistic approach, including physical, psychosocial and existential needs. It is unknown whether patients with different diagnoses and expected life spans will find these questions helpful in consultations about palliative care needs. A particular point of interest is the incorporation of the European Organization for Research and Treatment of Cancer Quality of Life Questionnaire Core 15 Palliative Care (EORTC-QLQ-C15-PAL) instrument, developed to assess health-related quality of life among cancer patients receiving palliative care [17]. In Denmark, palliative care is divided into two levels: basic and specialist care and the EORTC QLQ-C15-PAL has been used for specialist palliative care in Denmark since 2010 [18]. While the questionnaire for assessing palliative needs was already in use at the specialised level, there was a recognized need for a comprehensive tool applicable to non-specialised (basic) palliative care settings. To address this gap, PRO-Pall was developed as a PROM tailored for use across different healthcare settings. However, PROMs developed for specialised palliative care cannot automatically be applied to non-specialised palliative care settings To address this limitation, the PRO-Pall questionnaire was developed as an expanded version of the EORTC-QLQ-C15-PAL, incorporating additional elements to suit a broader patient population, including those with heart, lung, and kidney diseases, and cancer.

Therefore, this study aims to explore patients’ experiences of responding to the PRO-Pall and using it in consultations about palliative care needs in non-specialised palliative care settings.

## Methods

This national, multicentre mixed-methods study is part of a larger feasibility test of the PRO-Pall under the Danish Health Data Authority [15, 19, 20]. The development of PRO-Pall was a co-designed, multidisciplinary effort by a working group consisting of approximately 40–50 members including patients, patient representatives and professionals from various medical specialities [14, 19–21]. Professionals included regional physicians and nurses specializing in oncology, cardiology, nephrology, and pulmonology, and professionals from general practice, municipal healthcare, and quality assurance teams. Patient organization representatives, including those from the Danish Cancer Society, Heart Association, Kidney Association, and Lung Association, played an active role in the development process [14, 19–21]. Their combined expertise ensured that PRO-Pall addressed the needs of both patients and healthcare providers in basic palliative care settings [14, 20, 21]. The researchers of the present study participated in evaluating the PRO-Pall in a research partnership, which secured the right to analyse and publish the data independently of the authority.

This study employs a convergent parallel mixed methods design, as outlined by Creswell and Clarke [22]. This approach involves the simultaneous collection and independent analysis of both quantitative and qualitative data, which are later integrated to provide a comprehensive understanding of the research question. The design was chosen to allow for a multi-faceted exploration of patients’ experiences with PRO-Pall, offering both statistical insights and contextual depth. The study consists of two key components: a quantitative cross-sectional evaluation survey and exploratory qualitative interviews. Both data types were collected and analyzed concurrently, and integration was conducted using a side-by-side comparison approach in the discussion section. [23]. The integration of quantitative and qualitative findings enables a more robust understanding of the research questions by validating and expanding upon the insights gained from each method.

### The PRO-Pall questionnaire

The PRO-Pall questionnaire is intended to be a supportive tool to facilitate better palliative care. The PRO-Pall tool assesses physical, psychosocial, and existential issues through a total of 24 items, whereof, 15 from the EORTC QLQ-C15-PAL [17]. This tool has undergone extensive psychometric testing and is widely used in palliative care across different countries and languages [24]. Three additional items related to oedema, loneliness, and intimacy/sexual health were drawn from the EORTC Item Library to complement the core questionnaire [25]. These library items have undergone preliminary psychometric evaluation by the EORTC Quality of Life Group [25].

To further capture key aspects of patient experiences, five newly developed items address sore and dry mouth, social support, practical support, and existential problems [19]. Finally, the questionnaire was supplemented with the Write In Three Symptoms/Problems questionnaire, where respondents can note, in free text, up to three symptoms/problems not previously mentioned in the PRO-Pall (Attachment 1) [20, 26]. A detailed description of the development and structure of the questionnaire, content and user test report can be accessed (in Danish) via the Danish Health Data Authority’s official website [15]. Before pilot testing the PRO-Pall, a cognitive and linguistic analysis of the questionnaire was conducted by three clinical psychologists. They assessed the questions, response options, and overall comprehensibility based on psychological theory, methodology, and experience [27]. This analysis identified potential areas where respondents might face difficulties, which were subsequently tested in a content validation phase with fifteen patients, including patients with COPD, heart disease and cancer from multiple palliative care departments [27]. The content validation aimed to identify comprehension issues or other concerns related to the questionnaire. While minor comprehension issues were noted, no significant changes were deemed necessary. Regarding psychometric evaluation, no formal statistical validation (e.g., reliability or validity testing) was conducted on the PRO-Pall in this study. However, the cognitive and content validation processes ensured that the questionnaire was understandable and relevant for the target population [27]. This was a deliberate methodological decision, as the present study aimed to explore patients’ experiences with the tool in a qualitative and practical context, rather than to assess its psychometric properties. However, a separate study focusing on psychometric validation—including reliability and construct validity—is currently underway (unpublished data).

The participating sites could choose whether to have the patients complete the PRO-Pall questionnaire electronically or on paper. For electronic distribution, the sites used existing local/regional IT systems.

### Evaluation survey

The data on the evaluation survey were collected from one research clinic, three municipalities, and five hospitals across Denmark. Patients were asked to complete an evaluation survey after completing the PRO-Pall questionnaire and after being in the consultation. The evaluation survey was paper-based with 11 questions and free text fields (Attachment 2) [19]. Questions focused on e.g. the relevance of the PRO-Pall questions, perceived level of difficulty of responding and understanding, use of the questions in the consultation, and whether the PRO-Pall questionnaire contributed to preparation for consultation. Responses were entered into a REDCap database. Data were analysed descriptively as numbers (n) and proportions of responses (%).

### Semi-structured individual interviews

Each participating site (Table 1) recruited 1–3 patients for qualitative semi-structured interviews. However, one municipality administered the PRO-Pall to nursing home patients who were too weak to participate in a research interview. A purposeful sampling strategy was used to obtain a variation regarding gender, age, marital status and level of education. The individual semi-structured interviews focused on the patients’ experiences answering the PRO-Pall questionnaire and using the answers during the consultation with healthcare professionals (interview guide in Attachment 3). Patients were interviewed in Danish over the telephone, alone or in the presence of a family caregiver, based on the patient’s preference. The interviewers did not know any of the patients beforehand. If the patients preferred not to communicate via telephone, face-to-face interviews were conducted in the hospital units or at home.

The qualitative interviews were audio-taped and transcribed verbatim. The first author conducted thematic analysis using Braun and Clarke’s six-step framework for thematic analysis, a widely used approach in qualitative research that involves familiarization with the data, generating initial codes, searching for themes, reviewing themes, defining and naming themes, and producing the final report [28]. To ensure the rigor of our qualitative findings, we followed Braun and Clarke’s six-phase framework for thematic analysis, maintaining reflexivity, using peer debriefing, and triangulating qualitative and quantitative data. While data saturation was not the primary focus, themes remained consistent across participants, reinforcing the trustworthiness of our results. The software NVivo was used to support data management and coding, ensuring a structured and transparent analytical process. To enhance the credibility and trustworthiness of the findings, themes were discussed with one co-author (MR) and agreed upon after discussion. The quotations used in this article were translated by a multilingual translator service and checked by native Danish co-authors to ensure that the meaning did not change during translation.

### Setting and participants

**Fig. 1:**
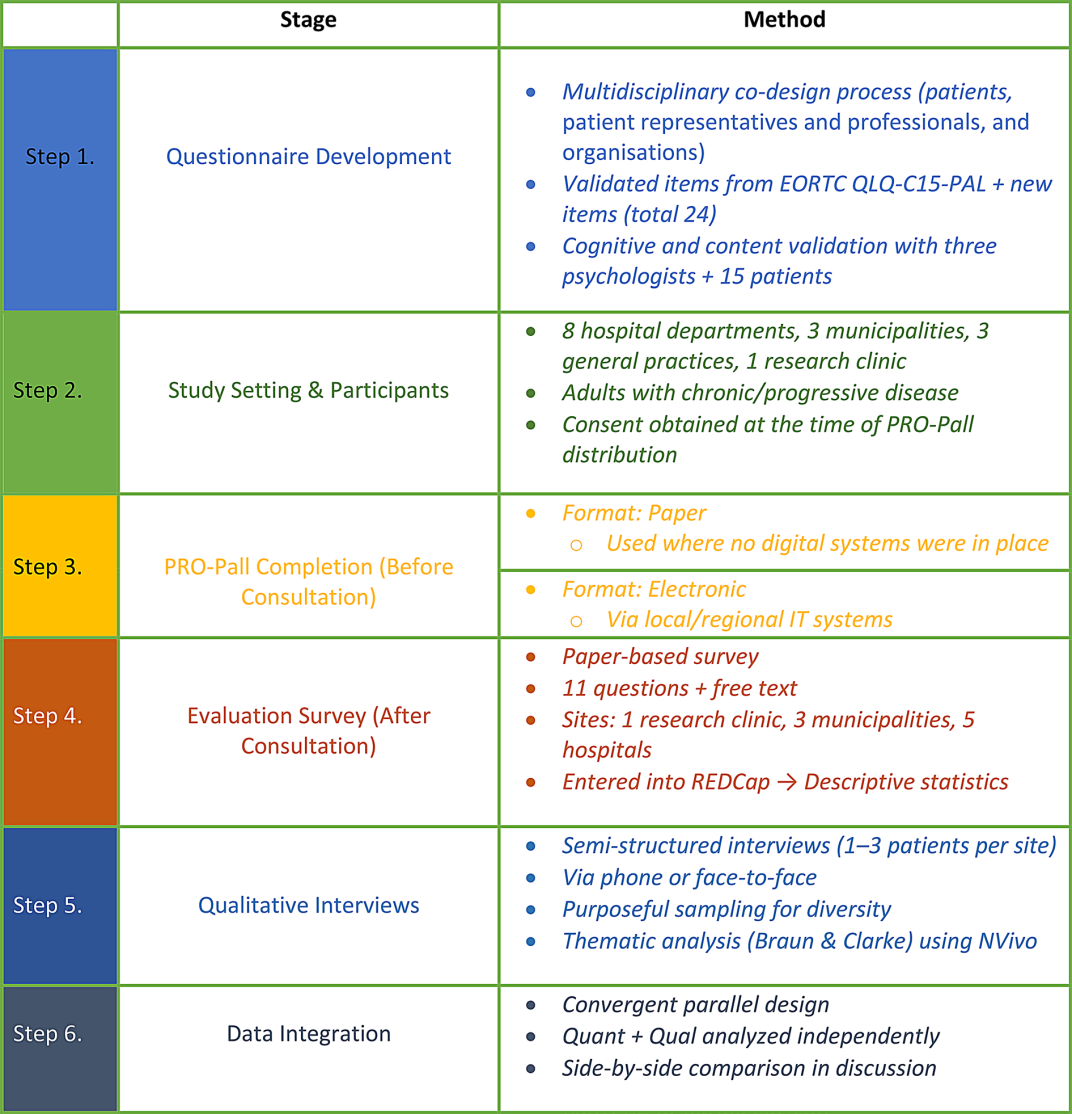
Flowchart of PRO-Pall Feasibility Study Design, Data Collection and Analysis

The feasibility test took place from November 2021 to October 2022 at three general practices, three municipalities, a research clinic and eight departments at five hospitals, including cardiology (1), pulmonary medicine (3), nephrology (1), oncology (1), and surgery (2). Adult patients with chronic or progressive cancer, heart, lung, or kidney disease were eligible to participate [19]. They also had to be cognitively capable of answering the questionnaire, which was assessed by a healthcare professional [19]. Patient consent was obtained during questionnaire distribution, in line with local practices and care processes, such as upon admission or at the start of ambulatory treatment, when they were first contacted about PRO-Pall data collection. The distribution of patient responses from these different settings is provided in Table 1. This approach ensured representation fromdiverse care environments. The general practices did not distribute the PROM but used PRO data from a collaborating municipality. Step by step data collection process and study desing are shown in Fig. 1.

### Ethical considerations

This study followed the Declaration of Helsinki [25], with informed written consent obtained from all participants, and national requirements for health science research were followed. The Scientific Ethics Committees for the Region of Southern Denmark assessed that according to Danish legislation, this study was not subject to ethical notification (j.nr. 20222000-06). The study was registered with the Region of Southern Denmark data protection agency (j.nr. 22/4403). Data were stored in OPEN– Open Patient Data Explorative Network [29] license nr. OP_1551.

## Results

### Quantitative data– evaluation survey

A total of *n* = 270 patients completed the evaluation survey, of which *n* = 126 (47%) were men. The characteristics of respondents are shown in Table 1.

**Table 1:** Characteristics of the 270 patients who responded to the evaluation survey

Characteristic		Respondents *n* (%)
Age	< 50 years	14 (5)
	50–59 years	40 (15)
	60–69 years	65 (24)
	70–79 years	101 (37)
	> 80 years	50 (19)
Sex	Male	126 (47)
	Female	144 (53)
Sites	Hospital	182 (67)
	Municipality	43 (16)
	Research clinic	45 (17)
Diagnosis	Lung disease	98 (36)
	Heart disease	30 (11)
	Kidney disease	21 (8)
	Cancer	109 (40)
	Lung disease and cancer	4 (1)

**Fig. 2:**
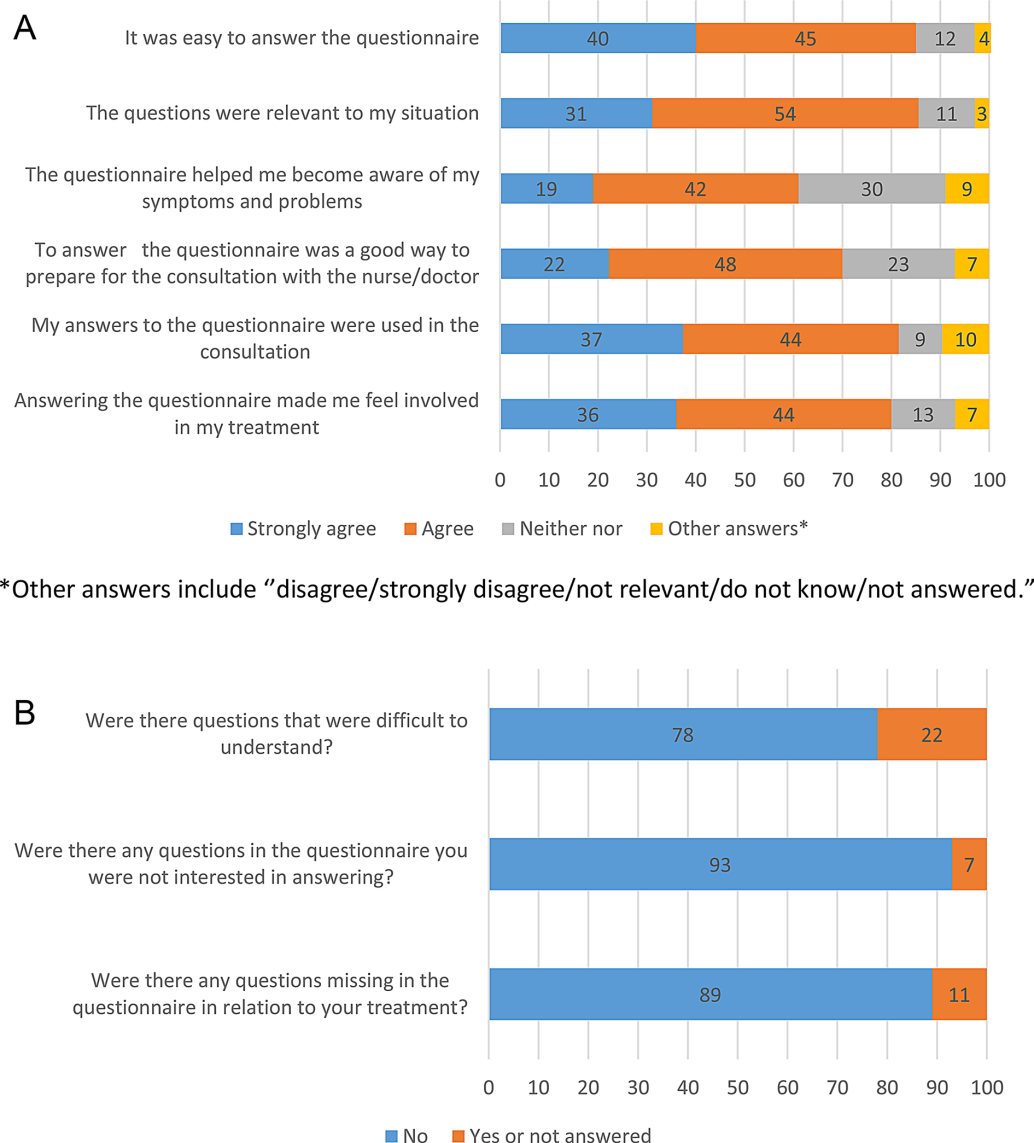
**a** and **b** Results of the evaluation survey n (%)

82% completed the PRO-Pall questionnaire on paper, and 79% spent 15 min or less completing it. They strongly agreed or agreed that the PRO-Pall helped prepare for the consultation, and 61% strongly agreed or agreed that they had been helped to become aware of their symptoms and problems. Further, 84% of the patients strongly agreed or agreed that the PRO-Pall answers had been used in the consultation, and 80% strongly agreed or agreed that responding to the PRO-Pall made them feel involved in their treatment. The proportion of missing answers was low (0–3%). Detailed results are presented in Fig. 2a and b.

### Qualitative data: interviews

Seventeen patients (10 women, age range 25 to 81 years, median age: 65 years; mean age 63 years; standard deviation: 14 years) were interviewed; thirteen interviews were conducted via telephone, and four were face-to-face. Four patients were from two municipalities, three from a research clinic, and the remaining ten were from four hospital departments—three from each of three departments and one from a fourth department. Three main themes were deduced from the data: experiences answering the PRO-Pall, experiences of using PRO-Pall responses in the consultation, and overall impression. Table 2 summarises the themes, followed by a more in-depth presentation.

**Table 2:** The themes and subthemes derived from the thematic analysis

**Experiences of answering the PRO-Pall**
• Motivation to fill out PRO-Pall
• Content and relevance of questions
**Experiences of using PRO-Pall responses in the consultation**
• Wider scope of dialogue
• Absence of consultation
**Overall impression**
• PRO-Pall– a tool for self-reflection
• Changing condition

### Experiences of answering the PRO-Pall

#### Motivation to fill out PRO-Pall

This theme captures the factors influencing participants’ willingness and engagement in completing the PRO-Pall. It includes intrinsic motivations, such as perceived benefits for their care, and external influences, such as encouragement for healthcare providers. A majority of patients experienced problems recalling the reason for answering the PRO-Pall. However, the patients perceived that filling out the questionnaire was was beneficial for themselves and additionally, they indicated that it could be beneficial for researchers and healthcare professionals.

*“If the doctors*, *nurses and others would benefit*, *then I am all for it.”* P-7.

*I completed it because research may influence the future. And then*, *hopefully*, *it can help others.”* P- 9.

Another motivation for filling out the PRO-Pall was to help other patients by providing information to the healthcare system.

“*Filling out the questionnaire makes you think of things you had not considered before. It is beneficial and could be used for something meaningful.*” P-12.

Patients expressed that they were motivated and willing to participate in filling out the PRO-Pall, hoping that PRO-Pall could identify their needs and wanted to help others. However, some of the patients lacked continuity in the disease trajectory: they were tired of completing too many questionnaires in their critical physical condition, as they often were perceived as repetitive, lengthy, time-consuming, and lacking follow-up. Therefore, patients were not always motivated to participate in filling out questionnaires.

“*It is very frustrating for the patient if you’ve spent time completing a similar questionnaire. I think it was a big problem. I can tell you that such questionnaires are pretty much the same. I don’t feel it’s targeted to me. I mean*, *we aren’t there. It’s not specifically something for my sake.”* P-5.

#### Content and relevance of the PRO-Pall questions

This theme reflects participants’ perceptions of whether the questions in PRO-Pall were meaningful, appropriate, and applicable to their condition. It includes discussions on the comprehensiveness, specificity, and clarity of the questions.

Overall, patients found the language of the questionnaire easy to understand. Therefore, most patients did not need external help filling out the questionnaire. Participants highlighted that the questionnaire’s content was neither harmful nor surprising. Regarding the length of the questionnaire and the time required to answer it, patients responded that it was reasonable.

*‘’The questionnaire was really easy to fill out. It was not difficult at all. I think it took me only five minutes. It could’ve been longer*, *but it was fine as it was.”* P − 6.

One patient praised the formulation of the questions, which had a positive impact:

“*I think the way the questions were formulated was very positive. I felt good after completing the questionnaire.‘’* P- 4.

Further, filling out PRO-Pall aided in preparing the patients for the consultation with healthcare professionals.

*‘’I always welcome such questionnaires before a consultation because there are some things you would’ve otherwise forgotten.”* P- 6.

However, one patient suggested adding more explanation on specific questions to understand the question’s meaning better:

“*I think there were some questions that were difficult to answer. An elaboration to what the question meant was lacking.”* P-15.

Others criticised it for being too general to identify specific needs. The patients who perceived the PRO-Pall as irrelevant found it challenging to relate to the questions. These patients mentioned that the questions did not seem to be developed to address the patient’s needs but were instead used to conduct research.

*‘’I found it hard to relate because the questions were not personal. They were too general. They were made for research and were not personal.”* P- 9.

Generally, patients perceived the PRO-Pall as a way of conveying information to healthcare professionals. By using PRO-Pall, the patients felt safe knowing that essential information reached all healthcare professionals involved in their care. Moreover, the patients felt that the answers could help healthcare professionals better understand their patients.

### Experiences of using PRO-Pall responses in the consultation

#### Wider scope of dialogue

This theme encompasses how the PRO-Pall responses contributed to a more open and expansive discussion during consultations. It includes instances where the tool helped patients express concerns, recall issues, or initiate conversations they might not have otherwise brought up.

Patients expressed the importance of a consultation after completing the questionnaire, which included a follow-up on their answers. This allowed patients to evaluate and elaborate on concerns related to their needs. The patients emphasised that the questionnaire structured the consultations to be more organised and focused on the primary problems. Thus, it became easier for patients to manage their symptoms, particularly around psychological issues, as described by a patient below:

*" Yes*, *there is a lot about the psychological aspects. First*, *he’s (the healthcare professional) good to talk to. He is also very good at helping you put things in order. So you go through both the development of your physical illness and also the psychological aspect (symptoms) of it*, *right.*” P-4.

The PRO-Pall contributed to improved communication between patients and healthcare professionals.

*“She (the nurse) was good at capturing my problems. Sometimes*, *one’s mood is not quite as good as it should be*, *and how am I supposed to deal with it when there is too much going on with the family and things like that? I think she understood that*, *and we got to talk about it because it was actually what concerned me the most.”* P-10.

Overall, the patients who perceived that their PRO-Pall answers were used in the consultation experienced that answering the questionnaire had a positive impact. It helped to build up a common understanding between patients and healthcare professionals, allowing the consultations to be broader.

#### Absence of consultation

This theme captures instances where PRO-Pall responses did not lead to discussions in the consultation. It includes participants’ experiences of their responses being overlooked or not integrated into the dialogue with their healthcare providers.The patients’ perspectives on the PRO-Pall questionnaire differed noticeably depending on whether or not their responses were discussed in the consultation. Five of the seventeen interviewees reported not discussing the PRO-Pall answers in the consultation. Notably, the patients who did not experience their answers being used in a consultation found that filling out questionnaires was not helpful overall.

*" I think every time you have to complete a questionnaire… you ask yourself*, *what will it be used for*, *and what do I get out of it? I hope you get something out of it. I don’t know what I get out of it*, *how much I get out of it.‘’* P-5.

A patient described the importance of follow-up after filling out the questionnaire:

” *I have no problem at all answering questionnaires. As long as someone follows them up. If you complete a questionnaire*, *and it is just put in a pile with fifty thousand others*, *and then one day*, *someone says shouldn’t we tidy up*, *I get annoyed. So that someone follows up*, *that’s very important.‘’* P-3.

One patient described that filling out the questionnaire in combination with the consultation was meaningful:

*‘’This usually happens when you feel bad*, *and someone asks how you are. Then you suddenly feel miserable*, *sad*, *or something. Then she (the nurse) picked it up*, *and we sat down and talked about the things that were concerning me. We didn’t go into the questionnaire too much*, *either. However*, *that was fine. In other words*, *the questionnaire alone wasn’t relevant.”* P-10.

### Overall impression

#### PRO-Pall– a tool for self-reflection

This theme refers to how participants perceived PRO-Pall as a means to reflect on their own health, symptoms, and needs. It includes how the tool helped them gain awareness of their condition, track changes, or articulate concerns.

Patients reported that the PRO-Pall contributed to person-centred care by focusing on the whole person. Further, some patients experienced that filling out the questionnaire aided their self-reflection around the disease and symptoms. For patients with an increase in their well-being, the PRO-Pall was used to reflect on their situation. Patients mentioned that filling out the PRO-Pall was like an eye opener to become aware of specific symptoms, which helped prepare the patient to discuss these with the healthcare professionals and eventually to symptom management or illness management in general.

*“Yes*, *it helps you*, *considering all physical changes. I became slightly aware that my illness was deteriorating. But it was fine because it confirmed what I felt was real.”* P-6.

Patients also mentioned how the PRO-Pall helped focus on the psychological aspects of their disease.

*" Completing the questionnaire helped about symptoms and mental feelings and things like that. I think it was great.”* P-11.

#### Changing condition

This theme captures participants’ recognition of their evolving health status as reflected through their PRO-Pall responses over time. It includes how completing the tool highlighted changes in symptoms, functional abilities, or emotional well-being.

Several of the interviewed patients, who were in different stages of their disease trajectory, found it challenging to describe the symptoms of their continuously fluctuating physical condition. According to the majority of patients, completing the PRO-Pall questionnaire earlier or later would have changed their answers since their symptom intensity varied.

*‘’One can say that it happens a lot. Big changes are happening. In one’s illness. I mean*, *there can be rapid changes*, *right?‘’* P-15.

Patients mentioned that physical symptoms that they experienced when filling out the questionnaire could be different compared to the consultation time, as it could take place up to 14 days after their responses. Thus, the intensity of the reported symptoms sometimes did not reflect their current situation. Several patients suggested that filling out the PRO-Pall questionnaire and the consultation should be closely linked:

*‘’In my experience*, *filling out the questionnaire as close to the interview as possible is important. If you got the questionnaire three weeks ago and filled it out immediately*, *the situation may have changed during those three weeks. As close to the next consultation as possible*, *that would be a good idea.”* P-3.

With few exceptions, the patients were in favour of answering the PRO-Pall again during their disease trajectory. However, the patient’s situation assessment determined how often they would like to fill out the PRO-Pall.

“*Frequency of completing it should be based on when it is relevant. Once is okay.”* P-12.

“The *questionnaire should be completed when needs change*, *not specifically every three or six months.*’’ P-2.

Patients were aware that their (palliative) care needs changed over time. Therefore, the PRO-Pall responses would no longer reflect their current status and would have scored differently if the questionnaire had reached them at a different point in the disease trajectory.

## Discussion

This study is the first to explore palliative care patients’ experiences using PRO-Pall within non-specialised palliative care settings in Denmark. This feasibility study showed the convenience of filling out the PRO-Pall and its relevance to patients with life-threatening cancer, heart, lung, and kidney diseases. The results may aid in overcoming perceived barriers mentioned by patients and facilitate using PROMs in clinical practice.

The evaluation survey and qualitative interviews indicate that the PRO-Pall questionnaire is easy to understand and reasonable in terms of length and time consumption. Contrary to our study results, a survey conducted within specialist palliative care units in Germany reported that most participants (73.7%) received support in completing PRO [30]. We found that using PRO-Pall as a basis for the consultation between patients and healthcare professionals facilitated discussions about palliative care needs, even though the patients did not explicitly express their needs as “palliative”. Thus, our findings are consistent with existing evidence on using PROMs in clinical practice, indicating that they may improve communication and be used integrated into consultations [7, 31, 32]. Further, similar to existing evidence [7, 32], our interview findings indicated the significance of PROMs in initiating discussion on patients’ psychological issues. Our study found that PRO-Pall provides a platform for a broader range of consultation topics between patients and healthcare professionals. A Cochrane review of PROMs used in clinical practice indicated that PROMs can provide moderate benefits for patient-provider communication [33]. While most patients in both the survey and the interviews felt their data were used in the consultation, importantly, our qualitative interviews with patients emphasised that filling out PRO-Pall was not meaningful unless PRO-Pall was subsequently incorporated into a consultation with healthcare professionals. The results of studies conducted in primary care [34] and among epilepsy patients [35] have consistently indicated similar findings. These findings are noteworthy and reinforce the importance of further research in this area. Both in the evaluation survey and during our interviews, patients expressed interest in filling out the PRO-Pall questionnaire again later in their disease trajectory. However, the perceptions of a relevant interval differed according to needs, as seen in previous studies [36, 37].

Our survey and questionnaire data indicated that a minor proportion of the patients did not experience a thorough follow-up on their PROM answers during the consultation. This suggests that some participating sites were better at implementing the PRO-Pall than others. Several factors can contribute to the lack of use of PROMs, such as poor patient health, lack of organisational support, low motivation among healthcare professionals, and insufficient local resources [3–5, 38, 39]. For example, implementing a new questionnaire might be challenging in the current workflow of the existing healthcare setting, adding a new step to the process that may take even more time. Previous studies have mentioned that training among healthcare professionals and adequate resources are essential to successfully implement PROMs for palliative care consultations [7, 35]. A significant amount of time and resources may be required from healthcare professionals to collect PROMs due to the cognitive and physical needs of palliative patients [9, 40]. Thus, addressing organisational barriers and providing appropriate training towards healthcare professionals has been suggested to achieve successful implementation [31, 40, 41]. Application of implementation models such as the Quality Implementation Framework may aid structured, evidence-based implementation efforts [42]. Of note, the timing of PRO-Pall introduction during the life-threatening illness trajectory may also be significant to patients, whether during an acute hospital stay, in an outpatient clinic, or combined with disclosing the diagnosis.

A key challenge identified in this study was that many patients did not perceive themselves as “palliative,” reflecting a broader issue in defining palliative care, particularly for non-cancer populations. This aligns with existing research highlighting that terminology impacts engagement with palliative care services [43]. The terminology “palliative” in a PROM may influence patient acceptance, and alternative phrasing that emphasizes symptom relief and quality of life—as is done in Denmark, where it is referred to as the “Questionnaire for Relief and Quality of Life” may facilitate broader implementation. Further research should explore how language influences patient and clinician engagement with palliative care assessments.

While the present study provides valuable insights into how patients perceive the relevance and content of the PRO-Pall questionnaire, the lack of psychometric analysis is a limitation. Further studies are needed to establish the tool’s reliability and validity through appropriate statistical methods, such as exploratory or confirmatory factor analysis and item response theory. We recommend psychometric validation as an essential next step in the development and implementation of PRO-Pall in clinical and research settings. Ensuring accessibility for cognitively impaired patients remains an important consideration. While the development group discussed the possibility of proxy reporting, no formal proxy version was created. Future work should investigate whether family-reported adaptations of PRO-Pall could be beneficial, particularly for patients with advanced dementia or other cognitive impairments.

Future research should explore refinements to PRO-Pall based on patient feedback, and evaluate implementation strategies in real-world healthcare settings. Ensuring that PRO-Pall is both practical and well-integrated into clinical workflows will be key to its long-term success in improving palliative care.Key strengths of this study include the mixed methods design and national sampling among patients with palliative care needs across Denmark. Conducting a survey and semi-structured interviews with patients provided a comprehensive understanding of PRO-Pall’s impact on patient-professional consultation. Limitations in the quantitative study include that we have no information on the selection of patients who received the questionnaire and of the non-respondents. Most participating sites were hospitals, which may have influenced the response format and patient recruitment. While the PRO-Pall questionnaire was available in both paper and electronic formats, practical constraints, including time limitations for healthcare professionals, contributed to the predominant use of the paper format. Limitations in the qualitative study include that it was difficult for a few patients to assess the relevance of particular PRO-Pall questions during the interview, as the interview was conducted up to several days after completing the PRO-Pall. The risk of healthcare professionals’ bias when recruiting patients should be considered, e.g., they may have been more inclined to recruit patients who were more positive towards PROMs and/or more resourceful. Finally, only those patients who had filled out the PRO-Pall were included. This may limit the representativeness of the included patients and the results in this study.

## Conclusion

This study contributes insight into patients’ experiences using PRO-Pall in non-specialised palliative care in various healthcare settings in Denmark. Patients found PRO-Pall to be an effective conversational tool in multiple ways. The results point to the importance of discussing patients’ answers in the consultation, as PRO-Pall otherwise risks becoming a documentation obligation rather than providing a way for patients to express their needs and concerns.

## Electronic supplementary material

Below is the link to the electronic supplementary material.

Supplementary Material 1

## Data Availability

The data for this study are stored in the Open Patient Data Explorative Network (OPEN) under license number OP_1551. Due to privacy and ethical considerations, the data are not publicly available. However, de-identified data can be made accessible upon reasonable request to the corresponding author and following the relevant Danish data protection regulations. The study followed the Declaration of Helsinki and was registered with the Region of Southern Denmark Data Protection Agency (j.nr. 22/4403).
