# Supplementary material for: Patients’ experiences of responding to a Patient-Reported Outcome Measure for palliative care: a mixed method study
Source: Qual Life Res. 2025 Jun 14;34(9):2651–62. doi: 10.1007/s11136-025-04006-w (PMC12432079; doi:10.1007/s11136-025-04006-w)
Supplement: Supplementary file 1 — Supplementary Material 1 [file 11136_2025_4006_MOESM1_ESM.docx]

Attachment 1: An overview of the items in the PRO-Pall tool.

The following table has previously been published in the article Darum Sørensen, Henriette, et al. "Using a Patient-Reported Outcome Measure to Assess Physical, Psychosocial, and Existential Issues in COPD." *Journal of Clinical Medicine* 13.20 (2024): 6200. <https://pubmed.ncbi.nlm.nih.gov/39458150/>

The translation from Danish to English has been performed by the research team, and the translation has not been psychometrically evaluated.

| Item number | Wording | Response categories | Type of symptom/ problem | Source |
| --- | --- | --- | --- | --- |
| 1 | Do you have difficulty going for a short walk outdoors? | Not at all  A little  Somewhat  A lot | Physical | EORTC-QLQ-C15 PAL |
| 2 | Do you have to lie in bed or sit in a chair during the day? | Not at all  A little  Somewhat  A lot | Physical | EORTC-QLQ-C15 PAL |
| 3 | Do you need help eating dressing, washing or going to the toilet? | Not at all  A little  Somewhat  A lot | Physical | EORTC-QLQ-C15 PAL |
| 4 | During the past week, were you short of breath? | Not at all  A little  Somewhat  A lot | Physical | EORTC-QLQ-C15 PAL |
| 5 | During the past week, have you had pain? | Not at all  A little  Somewhat  A lot | Physical | EORTC-QLQ-C15 PAL |
| 6 | During the past week, have you had trouble sleeping? | Not at all  A little  Somewhat  A lot | Physical | EORTC-QLQ-C15 PAL |
| 7 | During the past week, have you felt weak? | Not at all  A little  Somewhat  A lot | Physical | EORTC-QLQ-C15 PAL |
| 8 | During the past week, have you lacked appetite? | Not at all  A little  Somewhat  A lot | Physical | EORTC-QLQ-C15 PAL |
| 9 | During the past week, have you felt nauseous? | Not at all  A little  Somewhat  A lot | Physical | EORTC-QLQ-C15 PAL |
| 10 | During the past week, have you been constipated? | Not at all  A little  Somewhat  A lot | Physical | EORTC-QLQ-C15 PAL |
| 11 | During the past week, were you tired? | Not at all  A little  Somewhat  A lot | Physical | EORTC-QLQ-C15 PAL |
| 12 | During the past week, did pain interfere with your daily activities? | Not at all  A little  Somewhat  A lot | Physical | EORTC-QLQ-C15 PAL |
| 13 | During the past week, did you feel tense? | Not at all  A little  Somewhat  A lot | Psychosocial | EORTC-QLQ-C15 PAL |
| 14 | During the past week, did you feel depressed? | Not at all  A little  Somewhat  A lot | Psychosocial | EORTC-QLQ-C15 PAL |
| 15 | How would you rate your overall quality of life during the last week? | 7 point Likert scale ranging from ‘Very poor’ to ‘Excellent’ | Quality of life | EORTC-QLQ-C15 PAL |
| 16 | During the past week, did you have sore or dry mouth? | Not at all  A little  Somewhat  A lot | Physical | New item |
| 17 | During the past week, have you had swelling in any part of the body? | Not at all  A little  Somewhat  A lot | Physical | EORTC item library |
| 18 | During the past week, have you missed intimacy? (e.g., closeness, tenderness, sex) | Not at all  A little  Somewhat  A lot | Psychosocial | EORTC item library |
| 19 | During the past week, I felt lonely | Not at all  A little  Somewhat  A lot | Psychosocial | EORTC item library |
| 20 | Have you been worried about whether your role towards family or friends has changed? | Not at all  A little  Somewhat  Alot | Psychosocial | New item |
| 21 | Have you had thoughts about life or your situation that you need to talk about? | Not at all  A little  Somewhat  Alot | Existential | New item |
| 22 | Have the problems you have had in connection with your illness (such as financial, practical or personal) been taken care of? | Problems have been taken care of;  Problems have mostly been taken care of;  Problems have partly been taken care of;  Problems have by and large not been taken care of;  Problems have not at all been taken care of | Psychosocial | New item |
| 23 | Have you been able to share your feelings with your family or friends as much as you would like? | All the time  Most of the time  Sometimes  Rarely  Not at all | Psychosocial | New item |
| 24 | Have you had any other significant symptoms or problems not mentioned in the questions above? | No  Yes. Please list the most important (up to three) and indicate the extent to which you have had the symptoms or problems in the last week | Any | WISP |

Attachment 2: Evaluation survey - patients/citizens

This evaluation survey has been developed for use in Danish, and the English version is approximate and for information only.

| Social Security No:_______________ |
| --- |
| Feedback from the user about the questionnaire and use of answers in the consultation |

We are interested in your opinion on the questionnaire that you have answered. We ask you to answer all the questions. The answers will be used to improve the questionnaire and its use in clinical practice. [here may insert informed consent]

Which disease do you have?

__________________________________________________________________________________________

Your name: ________________________________________________________________________________

How did you fill in the questionnaire?

Electronic □ On paper □

How long did it take you to complete the questionnaire?

1-5 min. □ 6-10 min. □ 11-15 min. □ 16-20 min. □ 21+ min. □

|  | Questions | Answer | Elaborate if necessary answer here |
| --- | --- | --- | --- |
| 1. | It was easy to answer the questionnaire | □ 1 Strongly agree  □ 2 Agree  □ 3 Neither nor  □ 4 Disagree  □ 5 Strongly disagree |  |
| 2. | Were there questions that were  difficult to understand? | □ No  □ Yes, quite a few  □ Yes, several | If so, which ones? |
| 3. | Were there any questions in the questionnaire you were not interested in answering? | □ No  □ Yes, quite a few  □ Yes, several | If so, which ones? |
| 4. | Were there any questions missing in the questionnaire about  your treatment? | □ No  □ Yes, quite a few  □ Yes, several | If so, which ones? |
| 5. | The questions were relevant to my situation | □ 1 Strongly agree  □ 2 Agree  □ 3 Neither nor  □ 4 Disagree  □ 5 Strongly disagree |  |
| 6. | The questionnaire helped me become aware of my symptoms and problems. | □ 1 Strongly agree  □ 2 Agree  □ 3 Neither nor  □ 4 Disagree  □ 5 Strongly disagree |  |
| 7. | To answer the questionnaire was a good way to prepare for the consultation with the nurse/doctor | □ 1 Strongly agree  □ 2 Agree  □ 3 Neither nor  □ 4 Disagree  □ 5 Strongly disagree  □ Not relevant/don't know |  |
| 8. | My answers to the questionnaire were used in the consultation | □ 1 Strongly agree  □ 2 Agree  □ 3 Neither nor  □ 4 Disagree  □ 5 Strongly disagree  □ Not relevant/don't know |  |
| 9. | To answer the questionnaire  made me feel involved in my treatment | □ 1 Strongly agree  □ 2 Agree  □ 3 Neither nor  □ 4 Disagree  □ 5 Strongly disagree  □ Not relevant/don't know |  |

Feel free to write other comments here:

_____________________________________________________________________________________________________________________________________________________________________________________________________________________________________________________________________________________________________________________________________________________________

Thank you very much for your answer. Hand the complete form back to your healthcare professional.

Attachment 3: Interview guide for patients/citizens on the "Questionnaire on how are you"

This interview guide has been developed for use in Danish, and the English version is approximate and for information only

Introduction:

[notes]

Gender

Age

Place of residence

Marital status

Education level

| No. | Questions | Aim |
| --- | --- | --- |
| 1 | **Start** |  |
| 1.1 | Now, the actual interview starts.  It is important that you tell us about both the good and the bad sides so that we can learn and do better. | Focus in interview |
| 2 | **Introduction** | Aim |
| 2.1 | What is your disease?  Where are you in your treatment process?  With whom did you have the consultation about your answers to the questionnaire? | Background |
| 2.2 | Who told you about the questionnaire?  When did you answer the questionnaire?  What were you told the questionnaire would be used for?  Who did you imagine would use your answers? | Introduction  to PROMs.  Motivation for answering. |
| 2.3 | Where did you answer the form? (At home/on site)  Did you have someone to help you? | The context for the answer. |
| 2.4 | How do you feel about using IT/internet in connection with receiving treatment? | IT-experience |
| 3 | **The experience of answering the questionnaire** |  |
| 3.1 | How was it for you to answer the questionnaire?  Was it hard or easy? (on a scale from☹1-5☺ 1= very difficult 5= very easy)  (<5Why, what will it take for it to be easier for you?)  Approximately how long did it take you to answer the questionnaire?  What do you think about that?  What do you think about the length of the questionnaire? | Questionnaire burden and feasibility. |
| 3.2 | Were there any questions that were difficult to understand?  How relevant were the questions to you?  Were there any questions that were not relevant to you?  Were there any topics you particularly liked or did not mind asking about? | Relevance. |
| 3.3 | Did anything surprise you when you answered the questionnaire?  Did you become aware of anything special about your health when you answered the form? E,g,. Symptoms or changes in your life in connection with your disease? | Disease insight. |
| 3.4 | Did the questionnaire affect how you prepared for your interview?  Did the questionnaire affect your expectations for this interview? Can you say something about how the questionnaire affected the thoughts/feelings you had before your interview? (In a positive or negative direction?) | Preparation and expectations for the interview  Active participation.  Concern |
| 4 | **The consultation/** **consultation itself** |  |
| 4.1 | Do you think your health professional/health personnel had seen your answers on the questionnaire before you met?  Did your health professional/health personnel show you your answers to the questionnaire?  How (screen/paper)?  Were there topics that were noticed (focused on) that were not relevant for you to bring up, or were there topics that were not noticed but should have been?  *[if it is on the screen, continue with the following questions]*  Was there anything in that display on the screen that did not suit you/something you didn't recognise? | Use of PROMs in the consultation.  Validity of algorithm views. |
| 4.2 | Was there anything you mainly wanted to talk about during the consultation? Do you remember what? Did you talk about it?  Did you use your questionnaire answers in the interview? How? In which way?  How did you feel that the questionnaire affected your consultation? (common understanding, created distance, building bridge, irritation) | Both parties set the agenda. |
| 4.3 | Did your healthcare professional suggest any exceptional help, care, care or treatment for what you discussed? What? | Better treatment |
| 4.4 | How did you feel that filling out the questionnaire and using the answers in the consultation affected your sense of security?  Overall, how did you experience the quality of the consultation? Do you feel the use of your answers affected the quality of the consultation? | Experiencing the value of PROMs in the consultation itself. |
| 5 | **Assessment of specific questions in the questionnaire** |  |
| 5.1 | Were there any questions that were difficult to understand? |  |
| 5.2 | Were there questions in the questionnaire that you were not interested to answer? |  |
| 5.3 | Do you remember how you felt about answering questions?  1. About your role towards your family/friends,  2. About your thoughts about life  3. Whether you are lonely  4. Whether you have missed intimacy?  5. About practical problems  6. About sharing thoughts and feelings with family and friends |  |
| 5.4 | Do you have any suggestions for changes? | Suggestions for revisions |
| 6 | **Conclusion** |  |
| 6.1 | Overall, how do you think it was to use the questionnaire in connection with your consultation? That is, to answer it and then display the answers in the consultation itself? | Overall assessment |
| 6.2 | What is the most positive thing about using the questionnaire in your treatment? | Positive |
| 6.3 | Has something been wrong? | Negative |
| 6.5 | Would you be interested in using the questionnire in the future for your consultations?  Why/why not?  How often do you think it is relevant? (weekly, monthly?) | Future use |
| 6.6 | Do you have anything else you want to add?  Is there something you think is essential in connection with the questionnaire that we have not talked about? | Closing |
|  | Thank you very much for the help! |  |
